# Fermented Tea as a Food with Functional Value—Its Microbiological Profile, Antioxidant Potential and Phytochemical Composition

**DOI:** 10.3390/foods13010050

**Published:** 2023-12-21

**Authors:** Karolina Jakubczyk, Łukasz Łopusiewicz, Joanna Kika, Katarzyna Janda-Milczarek, Karolina Skonieczna-Żydecka

**Affiliations:** 1 Department of Human Nutrition and Metabolomics, Pomeranian Medical University in Szczecin, 24 Broniewskiego Street, 71-460 Szczecin, Poland; joanna.kika@pum.edu.pl (J.K.); katarzyna.janda.milczarek@pum.edu.pl (K.J.-M.); 2 Center of Bioimmobilisation and Innovative Packaging Materials, Faculty of Food Sciences and Fisheries, West Pomeranian University of Technology, 35 K. Janickiego, 71-270 Szczecin, Poland; lukasz.lopusiewicz@zut.edu.pl; 3 Department of Biochemical Science, Pomeranian Medical University in Szczecin, 24 Broniewskiego Street, 71-460 Szczecin, Poland; karolina.skonieczna-zydecka@pum.edu.pl

**Keywords:** kombucha, green tea, black tea, polyphenols, chemical composition, acetic acid, microbiological composition

## Abstract

Kombucha is a fermented tea drink produced by a symbiotic culture of bacteria and yeast, known as SCOBY. Its base has traditionally been black tea, which has been recognized for its health-promoting properties, particularly its antioxidant activity based on its high content of pol-yphenolic compounds. A number of previous studies have demonstrated the equally favourable biochemical and phytochemical composition of green tea. The aim of this study was to analyse and compare the basic biochemical composition, microbiological composition and antioxidant properties of black and green tea-based Kombucha. The green tea-based Kombucha showed a quantitatively more abundant microbial composition (Lactic Acid Bacteria, *Acetobacter* sp., Yeast), a higher reducing potential (FRAP—4326.58 Fe(II)µM/L) and a higher content of total polyphenols (23.84 mg GAE/100 mL, reducing sugars (3212.00 mg/100 mL) as well as free amino acids (849.00 mg GLY/mL). Kombucha made from black tea, on the other hand, showed a higher anti-oxidant potential (1.17 Trolox (mM) TEAC), neutralising the DPPH radical at 94.33% and ABTS at 97.74%. It also had a higher level of acetic acid (0.08 g/100 mL). Green tea kombucha had a higher scavenging capacity of 90.6% for superoxide radical (O_2_^−^) and 69.28% for hydroxyl radical (·OH) than black tea kombucha. In the present study, both kombucha drinks tested were shown to be source of potent antioxidants. In addition, green tea, as a kombucha base, has proven to be as beneficial a raw material that will provide full nutritional and health-promoting values as traditional kombucha.

## 1. Introduction

Kombucha is a fermented tea drink made with a symbiotic culture of bacteria and yeast, known as SCOBY, which generally consists of acetic acid bacteria, lactic acid bacteria and yeast (*Acetobacter xylinum*, *Gluconobacter* sp., *Saccharomyces cerevisiae*) [1,2]. Historically, kombucha originated in China, where it has been an established health-promoting drink for thousands of years. However, it owes its current name to a Korean doctor—Kombu—who used it for therapeutic use. Subsequently, kombucha became popular in Japan and expanded from Asia to Europe [3,4].

Kombucha is prepared by adding sugar (10%), inoculum from a previous fermentation (10%) and SCOBY to tea. The SCOBY added to the sweetened tea initiates fermentation, which results in the production of various new bioactive compounds. Fermentation is carried out at room temperature (20 to 25 °C) over a period of 7–14 days. Various types of tea can be used to make kombucha, whether green tea or fermented tea, e.g., red, black or yellow. However, black tea and white sugar (sucrose) are considered to be the traditional and at the same time the best ingredients for the correct composition of the finished beverage and its health-promoting effects. The taste of a tea drink is described as sour, slightly fruity and slightly effervescent [5,6,7].

Studies of kombucha have proven its antimicrobial, antioxidant, antidiabetic, cholesterol-lowering, immune system-supporting and liver detoxification-stimulating properties [8]. However, to date there are no clinical studies available to confirm the health-promoting properties of this beverage. Minerals mainly derived from tea (potassium, manganese, fluoride ions), vitamins (E, K, B), amino acids (especially theanine, a glutamine derivative), as well as other compounds formed by numerous reactions during tea fermentation have been found in kombucha drinks. When polyphenolic compounds are oxidised, they are converted into new molecules [8,9].

Various parameters influence the properties and composition of kombucha, including the type of tea used, the amount and type of carbohydrate source used, fermentation time, SCOBY composition or temperature [1,10]. Despite the rising popularity of consumption of this beverage, there is still incomplete information on the effect of different fermentation parameters or type of tea on properties or composition. One of the factors that can determine both composition and properties may be the type of tea.

Inappropriate lifestyles, intense exercise, stress and environmental pollutants are all factors that contribute to the excessive production of reactive oxygen species. Imbalance caused by free radicals leads to oxidative stress and damage to structures in the human body [11]. Diseases that may be underlain by free radical-related disorders are mainly atherosclerosis, neurodegenerative diseases such as Parkinson’s and Alzheimer’s disease or even obesity. In order to maintain a balance between the production and removal of reactive oxygen species, readily available sources of antioxidants are being sought [2,11]. The main and most widespread antioxidants are vitamins E, A and C and polyphenolic compounds [11]. Sources of antioxidants are primarily sought in natural plant materials. Antioxidants are present in many readily available sources such as tea, coffee, fruit, vegetables, spices and herbs. They supplement the daily diet, helping to maintain health.

There are limited works analyzing and comparing the detailed biochemical composition, phytochemical composition, antioxidant potential, and microbial composition in kombucha with different base raw materials. Therefore, the aim of the present study was to analyse and compare the basic biochemical composition, microbiological composition and antioxidant properties of black and green tea-based kombucha.

## 2. Materials and Methods

### 2.1. Material

The study material was Kombucha based on green and black tea available on the food market (Delikatna.bio, Gdynia, Poland). Kombucha from 3 different glass bottles, stored at 4 °C for each type, were included in the study. The entire experiment was performed in 9 replicates. The characteristics of the basic nutrients were taken from the product label provided by the manufacturer. (Table 1) The raw materials for kombucha preparation came from certified organic cultivation. Samples were kept refrigerated until analyses were performed. Prior to all biochemical analyses the samples were filtered through 0.22-µm nylon membrane filters (Sigma-Aldrich, Darmstadt, Germany). The obtained clear fluids were used for further analyses.

### 2.2. The Determination of Lactic Acid Bacteria, Acetobacter sp. and Yeast

The samples (1 mL) were collected and diluted with 9 mL of sterile buffered peptone water (Merck, Darmstad, Germany), and serial dilutions were prepared [12]. Lactic acid bacteria counts were determined on MRS (de Man, Rogosa and Sharpe) medium (Merck, Darmstad, Germany) after incubation at 37 °C under anaerobic conditions for 72 h, whereas *Acetobacter* bacteria were assayed on Acetobacter Agar (HiMedia, Mumbai, India), after incubation at 37 °C under aerobic conditions for 48 h. Yeast counts were determined on Rose Bengal Agar at 25 °C for 72 h [13]. The enumeration of microorganisms was performed in triplicate (by counting plates with 30–300 colonies) and the viable cell counts were expressed as CFU/mL of the samples.

### 2.3. The Determination of Reducing Sugars Content (RSC)

The RSC (Reducing Sugars Content) was determined by the DNS (3,5-dinitrosalicylic acid) method as described elsewhere [12]. Briefly, 1 mL of each sample was mixed with 1 mL of 0.05 M acetate buffer (pH 4.8) and 3 mL of DNS reagent, then shaken vigorously. The mixtures were incubated in boiling water for 5 min, and cooled at room temperature (20 to 25 °C). The absorbance value was measured at 540 nm using a microplate reader (Synergy LX, BioTek, Winooski, VT, USA) by placing the samples in a 96-well microplate. Glucose (0.01–10 mg/mL) in acetate buffer was used for the calibration curve.

### 2.4. The Determination of Total Free Amino Acids

Total free amino acids (TFAA) were analysed following previously described methodology using a Cd-ninhydrin reagent [13]. Exactly 1 mL of extracts were mixed with 2 mL of a Cd-ninhydrin reagent. The samples were shaken, heated at 84 °C for 5 min, cooled in ice water and the absorbance at 507 nm was determined by the microplate reader (Synergy LX, BioTek, Winooski, VT, USA). The results were expressed as mg glycine (Gly) per mL of sample with respect to the standard curve including the dilution factor. The standard curve was first prepared using glycine.

### 2.5. The Determination of Total Polyphenols Content (TPC)

Determination of polyphenols was performed according to method using the Folin-Ciocalteu reagent [14]. Absorbance at 765 nm was measured (8453UV, Agilent Technologies, Santa Clara, CA, USA). All assays were performed in triplicate. The content of polyphenols was determined from the calibration curve using gallic acid (GAE) as the reference standard (0–200 mg/L of gallic acid).

### 2.6. The Determination of the Total Flavonoids Content (TFC)

Determination of total flavonoids content was performed according to the Pękal and Pyrzynska [15] and Hu et al., methods [16]. Different concentrations of flavonoids were used in the plotting of the standard calibration curve. The content of flavonoids was determined from the calibration curve using rutin equivalent as the reference standard (0–120 mg/L of rutin equivalent). Absorbance at 510 nm was measured (8453UV, Agilent Technologies, Santa Clara, CA, USA). All assays were performed in triplicate.

### 2.7. The Determination of pH

The pH of beverages was determined by a pH meter (SCHOTT Instruments; SI Analytics Mainz, Germany).

### 2.8. The Determination of Acetic Acid

Acetic acid (AA) was analysed by High Performance Liquid Chromatography (HPLC) using a1200 series HPLC connected to a 1100 series RI detector (Agilent Technologies, Santa Clara, CA, USA) with a Rezex ROA-Organic Acid H+ (8%) column (Phenomenex, Torrance, CA, USA). The column was eluted with a degassed mobile phase containing 5 mM H_2_SO_4_, pH 2.25 at 60 °C with a flow rate of 0.5 mL/min for 30 min per sample [17,18]. The results are shown in g/100 mL.

### 2.9. Antioxidant Activity by the DPPH and TEAC Methods

The antioxidant activity of samples was measured with the spectrophotometric method using synthetic radical DPPH (2.2-diphenyl-1-picrylhydrazyl, Sigma, Poznań, Poland) according to Brand-Williams et al. [19] and Pekkarinen et al. [20]. The spectral absorbance was immediately measured at 518 nm (8453UV, Agilent Technologies, Santa Clara, CA, USA). All assays were performed in triplicate. The results are shown in % of DPPH radical inhibition and in Trolox equivalent as the reference standard. The range of Trolox concentrations used to run the calibration curve: 1, 0.6, 0.2, 0.12 µM Trolox.

Antioxidant potential (antioxidant activity, inhibition) of tested solutions has been expressed by the percent of DPPH inhibition, using the following formula:

(1) $$\%\;inhibition=\frac{A_{0}-A_{s}}{A_{0}}\;\times \;100$$

where:

A_0_—absorbance of DPPH solution at 518 nm without tested sample
A_s_—absorbance of DPPH solution at 518 nm with tested sample
Total antioxidant potential by ABTS method

Antioxidants reduce ABTS^•+^ (oxidised form) to colourless ABTS (reduced form). The decrease in absorbance is a measure of the antioxidant content of the test material. Solution of ABTS in 96% ethanol, and 0.1 mL of the test sample were introduced into the vial. The prepared solution after mixing was placed for 6 min in a dark place. Ethanol was used as a reference solution and ABTS solution for A_0_. Before the measurement, the vial contents were thoroughly mixed and poured into cuvettes. The spectral absorbance was immediately measured at 734 nm. All tests were performed in triplicate.

Antioxidant activity has been expressed by the percentage of ABTS inhibition, using the formula:

(2) $$\%\;\mathrm{inhibition}=\frac{A_{0}-A_{s}}{A_{0}}\;\times \;100$$

where:

A_0_—absorbance of ABTS solution without the tested sample,
A_s_—absorbance of ABTS solution with the tested sample.

### 2.10. The Determination of the Ferric Iron Reducing Antioxidant Power (FRAP) Method

The FRAP method, used to determine the total reduction potential, which also means the antioxidant properties of tested ingredient, is based on the ability of the test sample to reduce Fe^3+^ ions to Fe^2+^ ions. The FRAP unit determines the ability to reduce 1 micromole Fe^3+^ to Fe^2+^ according to Benzie and Strain [21,22]. Absorbance at 593 nm was measured (8453UV, Agilent Technologies, Santa Clara, CA, USA). All assays were performed in triplicate. The ferric ion reducing antioxidant power was determined from the calibration curve using Fe(II)/L as the reference standard (0–5000 µM Fe(II)/L).

### 2.11. The Determination of Superoxide (O_2_^−^) Scavenging Activity

Superoxide (O_2_^−^) scavenging activity was carried out based on pyrogallol oxidation inhibition following methodology described elsewhere [13]. Three mL of 50 mmol/L (pH 8.2) Tris-HCl buffer were mixed with 1 mL of the samples. These mixtures were mixed with a pyrogallol solution (0.3 mL, 7 mmol/L, preheated to 25 °C) and allowed to react for exactly 4 min, then 1 mL of 10 mmol/L of HCl was added to terminate the reaction, and absorbance was measured at 318 nm (microplate reader (Synergy LX, BioTek, Winooski, VT, USA).

### 2.12. The Determination of Hydroxyl (·OH) Scavenging Activity

The hydroxyl (·OH) scavenging assay was carried out based on method of Ye et al. [23]. One mL of the samples and 1.5 mL of orthophenanthroline solution (0.005 mmol/L) were mixed with 2 mL of phosphate buffer (pH 7.4, 0.05 mol/L). Then 1 mL of FeSO_4_ solution (0.0075 mol/L) was added and then mixed with 1 mL of H_2_O_2_ (0.1%), and finally supplemented with distilled water to a total volume of 10 mL. The reaction solution was kept at 37 °C for 1 h in darkness, then the absorbance was measured at 536 nm (microplate reader (Synergy LX, BioTek, Winooski, VT, USA). The orthophenatroline solution without H_2_O_2_ addition (replaced by 1 mL of methanol) served as a blank.

### 2.13. Statistical Analysis

In all the experiments, three samples were analysed and all the assays were carried out at least in triplicate. The statistical analysis was performed using MedCalc^®^ Statistical Software version 22.006 (MedCalc Software Ltd., Ostend, Belgium) and Microsoft Excel 2017. The results are expressed as mean values and standard deviation (SD) or median. To assess the differences between examined parameters, a Mann-Whitney test and a *t*-test was used. Correlations between parameters were determined using Spearman rank correlation coefficient. Differences were considered significant at *p* ≤ 0.05.

## 3. Results

The kombucha drinks tested proved to be a valuable source of polyphenols, with concentrations of 20.7 and 23.95 mg GAE/100 mL in black tea kombucha and green tea kombucha, respectively. The total flavonoid content was significantly higher for traditional kombucha, at 138.12 mg RE/100 mL of beverage, compared to 98.2 mg RE/100 mL for green tea kombucha. The black tea kombucha exhibited a low pH (2.69) and high vitamin C content (27.62 mg/100 mL). The pH value of kombucha green tea was 2.99, significantly higher than that of traditional kombucha. The acetic acid content was non-significantly higher in the traditional kombucha and was 0.08 g/100 mL compared to the other type of kombucha tested, where acetic acid was determined at 0.03 g/100 mL. The green tea kombucha, on the other hand, was characterised by a significantly higher content of reducing sugars (3212 mg/100 mL) and total free amino acid content (849 mg GLY/mL). All results obtained relating to the biochemical composition of kombucha are presented in Table 2 and Table 3.

Given the proven antioxidant effects of kombucha and various types of tea, selected kombucha drinks made from green and black tea were tested to identify equally beneficial uses for other types of tea. The antioxidant potential depending on the method chosen for its determination was 3495 Fe(II)µM/L (FRAP) and 1.17 mM Trolox/L (TEAC) in black tea kombucha. The percentage of radical inhibition in this beverage was 94.33% (DPPH) and 97.36% (ABTS). The percentage superoxide radical scavenging activity (O_2_^−^) of the traditional kombucha was 90.03%. while the hydroxyl radical (∙OH) scavenging activity was lower at 67.23%.

Green tea kombucha had a significantly higher scavenging capacity for superoxide radical (O_2_^−^)—90.6% and hydroxyl radical (–OH)—69.28% than black tea kombucha. Its antioxidant potential was 91.26% and 95.6% for the neutralisation of the DPPH and ABTS radicals. The reduction potential determined by the FRAP method was found to be significantly higher than that of black tea kombucha and was 4326.58 Fe(II)µM/L. In contrast, the antioxidant activity of green tea kombucha was significantly lower than that of black tea and was determined to be 1.14 mM Trolox/L.

The results relating to the antioxidant properties of kombucha are presented in Table 4.

Depending on the SCOBY used the microbiological composition of the final product may vary. In order to identify the microorganisms present in the beverages tested, quantification of *Acetobacter* sp. Lactic Acid Bacteria and Yeast was carried out. Kombucha prepared on the basis of green tea, according to the results, contained a significantly more abundant quantitative microbial composition than kombucha based on black tea. The results are summarized in Table 5.

## 4. Discussion

Fermented beverages are becoming increasingly popular, as consumers perceive fermentation as a natural method of food preservation, and the products themselves are valued for their health-promoting properties, which are enhanced by the fermentation process [17]. Kombucha as a fermented tea drink is being consumed increasingly in Europe, despite being of Asian origin [24]. It is prepared primarily on the basis of black tea, however, other kombucha variants, such as those made from other types of tea, such as green, white or red tea and enriched with fruit or other flavours are increasingly popular on the food market [25,26]. Although, kombucha is well studied in terms of its microbial composition and its antimicrobial properties, there is a lack of research on the different base types of teas and their composition and health-promoting properties. For this reason, one of the most popular types, in addition to traditional black tea—green tea—was selected for our study, and the microbiological composition, phytochemical and antioxidant potential were checked depending on the type of tea chosen for Kombucha preparation.

Tea, which is one of the main ingredients in the kombucha drink, is abundant in polyphenols, which include theaflavin and thearubigin. The polyphenols present in tea are indirectly responsible for the final antioxidant activity of kombucha [6,9]. Chakravorty et al. [27] and de Noronha et al. [28] have noted, that during fermentation, the amount of polyphenols increased. The increase in polyphenolic compounds may be related to a number of reactions occurring during tea fermentation. e.g., during the oxidation of polyphenolic compounds by certain enzymes, catechins are formed, flavonoids and other compounds with health-promoting properties, including antioxidant properties [9]. Also microorganisms are capable of degrading polyphenols [29]. The catechins in tea can be broken down by bacterial and yeast activity to simpler molecules. thus increasing antioxidant power [9,30]. Based on our study, it can be concluded, that the total polyphenol content is dependent on the type of tea. Higher concentrations were recorded in kombucha made from green tea, whereas slightly lower for kombucha made from black, which is in the line with the results of other researchers [18,31]. The opposite results were obtained in the study by Cardoso et al. [32] where kombucha from black tea that showed higher levels of polyphenols (1.09 mg GAE/mL) than kombucha from green tea (0.7 mg GAE/mL). The total flavonoid content of black tea kombucha was 138.12 mg/L, which was the same as the results of other authors (126.7 mg/L). In contrast, in this work we obtained a 2-fold lower result for green tea kombucha (93.2 mg/L vs. 181.3 mg/L) [31].

Vitamin C has strong antioxidant activity due to its ability to effectively neutralize free radicals. During the fermentation process, the vitamin C content of beverages can increase [6,33]. Kaewkod et al. [18] observed higher vitamin C contents (0.61 g/L for green tea and 0.7 g/L for black tea) than in this study. However, it should be noted. that this vitamin is very unstable. It is estimated that 100 mL of kombucha based on black tea will provide 31% of the RDA for an adult male for this dietary component, while kombucha made from green tea will provide 30% of the RDA, so kombucha can be a good source of this component in the daily diet [34].

Kombucha, regardless of the type of tea used, is characterised by its high antioxidant potential [35,36]. In animal models, it was shown, that oral administration of kombucha tea to lead-exposed rats reduced lipid peroxidation and DNA damage, while increasing reduced glutathione levels and glutathione peroxidase (GPx) activity [37]. The determination of free radicals scavenging activity, which is hydroxyl (·OH) at 67.2–69.3% was higher in this study than observed by other researchers (44.8–52.3%) [9]. This work also determined the level of superoxide radical (O_2_^−^) neutralisation activity, which was 90% for traditional kombucha and 91% for green tea kombucha. This demonstrates the high antioxidant activity of the kombucha beverage tested, which, according to other reports, increases with the progress of fermentation [33]. In addition, the antioxidant potential measured by the DPPH method in the present study was determined at a significantly higher level for both teas (94.3% black tea and 91.3% green tea) than in the study by Malbaša et al. [33] (45% and 60%) and Jakubczyk et al. [31] (61.0% and 88.2%). Also in the above-mentioned works, the relationship of higher potential in kombucha from black tea was not preserved, as observed in the present study. A similar result (97.7% and 96.3%) was obtained for the ABTS radical scavenging assay. The reduction potential measured by the FRAP method was determined at a significantly higher level in our work than that of other authors. Jakubczyk et al. [31] observed a reduction potential in green tea kombucha of 3172.9 µM Fe(II)/L, where in this study its level was 4350.6 µM Fe(II)/L. Also, a significant difference was observed with respect to the results of kombucha made from traditional tea, with our result being 3495 µM Fe(II)/L, while the authors’ comparison was 1573.9 µM Fe(II)/L. In the study by Chakravorty et al. [27] an increase in the antioxidant potential of Kombucha was observed compared to the base tea. It is therefore important to study the final product, not only the base raw materials. The present study showed, that the antioxidant properties of Kombucha depend on the type of base raw material.

An important parameter that changes during fermentation is the pH of the solution and the acidity i.e., the organic acid content. The microorganisms contained in the SCOBY process the sugar and substances contained in the tea, producing a variety of metabolites. The fermentation of kombucha is a combination of three types of fermentation, which are: lactic fermentation, alcoholic fermentation and acetic fermentation. Lactic fermentation is responsible for the breakdown of glucose, which occurs because of Lactic Acid Bacteria metabolic activity. Initially there is an increase in reducing sugar. which can be attributed to the hydrolysis of sucrose to glucose and fructose by yeast. As fermentation continues, the yeast anaerobically uses the sugar to produce ethanol [9]. Gaggìa et al. [17] checked the content of glucose, fructose and sucrose in kombucha prepared from black, green and red tea on days 7 and 14 of fermentation. The content of complex sugars. i.e., sucrose, decreased during fermentation. while simple sugars (such as glucose) increased. In our study, the content of reducing sugars was higher in kombucha based on green tea, which may be related to the potentially longer fermentation process. The carbohydrate source used may also have had an impact as well. Alcoholic fermentation is another type of fermentation. Yeast, which is part of the beverage’s microflora, is responsible for metabolizing glucose into ethanol with the release of carbon dioxide. In the next stage, acetic acid bacteria (such as *Acetobacter* sp.) use ethanol as a substrate to produce acetic acid [8]. During fermentation, the alcohol content therefore decreases, while acidity increases and organic acids are produced. Acetic acid, which is the predominant organic acid present in the fermented solution. contributes to a decrease in pH from 5 even to 3 [9,38]. This is consistent with the results of the present study, as kombucha from black tea has a lower pH (2.69) than from green tea (2.99), while at the same time having a higher acetic acid content (0.08 g/100 mL vs. 0.03 g/100 mL). These values are within the consumer safety range (2.5–4.2 g/L) [32]. It should also be emphasised, that the pH of the solution and the presence of certain organic acids determine the growth of microorganisms and thus the chemical composition of the beverage [17]. Low pH and high acidity allow the growth of only those microbes, which are able to inhabit such extreme conditions and thus can provide some protection against undesirable microorganisms [39].

All of the microorganism species determined (Lactic Acid Bacteria, Yeast and *Acetobacter* sp.) were found in higher levels in kombucha green tea than in traditional kombucha tea, which may be related to both the different composition of the SCOBY starter (no manufacturer information available) and the type of base material. Thus, there is a need for further research to determine the optimal composition of SCOBY in terms of increasing the synthesis of compounds that exhibit health-promoting, particularly antioxidant, effects, while maintaining the flavor of the final product. In addition, it is worth extending the research on the impact of the choice of raw material from which the beverage is made on its final microbiological composition.

## 5. Conclusions

Kombucha (or fermented tea) has strong antioxidant properties. It exhibits high antioxidant potential, which can be attributed to its high content of polyphenols and flavonoids. The antioxidant activity, as well as the phytochemical composition of kombucha, varies depending on the type of tea used to prepare this beverage. Choosing a tea other than the traditionally used black tea and subjecting it to the fermentation process seems to be equally beneficial in terms of the health-promoting properties of the final product. However, there is a need for further research to possibly expand the range of health-promoting beverages.

## Figures and Tables

**Table 1:** Nutritional values in 100 mL of tested kombucha, claimed by the manufacturer.

Kombucha		Black	Green
ingredients	black tea *, cane sugar *, SCOBY culture		green tea *, concentrated apple juice *, SCOBY culture
Energy value	kJ/kcal	82/20	18/4
FAT	g	<0.1	<0.1
of which saturated fatty acids	g	<0.1	0
Carbohydrates	g	5	<0.5
of which sugars	g	5	<0.5
Protein	g	0.3	0
Salt	g	0.06	0
Ethanol	%	<1.2	<1.2

* from certified organic cultivation.

**Table 2:** Parameters of tested kombucha beverages.

		TPC	TFC	Vitamin C
Kombucha		GAE [mg/100 mL]	RE [mg/100 mL]	[mg/100 mL]
Black	Median	20.697 *	138.120 *	27.132
	IQR	20.531 to 21.087	135.073 to 140.185	21.450 to 34.258
Green	Median	23.949 *	93.203 *	27.174
	IQR	23.699 to 24.068	86.600 to 104.219	24.606 to 27.690

* *p* < 0.05 statistically significant difference in column; IQR—interquartile range.

**Table 3:** Biochemical composition of tested kombucha beverages.

		pH	Acetic Acid	Reducing Sugars Content	Total Free Amino Acid
Kombucha			acetic acid [g/100 mL]	[mg/100 mL]	[mg GLY/mL]
Black	mean	2.69 *	0.08	2842.00 *	710.00 *
	SD	0.11	0.00	0.02	0.02
Green	mean	2.99 *	0.03	3212.00 *	849.00 *
	SD	0.01	0.00	0.02	0.08

* *p* < 0.05 statistically significant difference in column.

**Table 4:** Antioxidant properties of the tested beverages.

		FRAP	TEAC	DPPH	ABTS	Superoxide Radical (O_2_^−^)	Hydroxyl Radical (·OH)
Kombucha		Fe(II)µM/L	Trolox (mM/L)	%	%	%	%
Black	Median	3495 *	1.168 *	94.33 *^,a^	97.358	90.03 *^,a^	67.23 *^,a^
	IQR	3389.500 to 3513.200	1.163 to 1.175	0.52 ^b^	97.262 to 98.413	0.02 ^b^	0.07 ^b^
Green	Median	4350.6 *	1.140 *	91.26 *^,a^	95.604	90.60 *^,a^	69.28 *^,a^
	IQR	4304.400 to 4369.800	1.117 to 1.146	1.74 ^b^	94.957 to 98.866	0.4 ^b^	0.02 ^b^

* *p* < 0.05 statistically significant difference in column; ^a^ Mean, ^b^ SD.

**Table 5:** Microbiological composition of the tested beverages.

Kombucha	Lactic Acid Bacteria	*Acetobacter* sp.	Yeast
Black	3.02 × 10^5^ CFU/mL ^a^	7.89 × 10^3^ CFU/mL ^a^	8.25 × 10^5^ CFU/mL ^a^
Green	1.06 × 10^6^ CFU/mL ^b^	1.06 × 10^4^ CFU/mL ^b^	8.50 × 10^5^ CFU/mL ^b^

^a,b^ Means with different lowercase letters in the same column are significantly different at *p* < 0.05.

## Data Availability

The data presented in this study are available on request from the corresponding author.
